# Sexual dysfunction in married women with Systemic Sclerosis

**DOI:** 10.11604/pamj.2014.17.82.3833

**Published:** 2014-02-02

**Authors:** Faten Frikha, Jawaher Masmoudi, Noura Saidi, Zouhir Bahloul

**Affiliations:** 1 Department of Internal medicine, Hedi Chaker Hospital 3029, Sfax, Tunisia; 2 Department of psychiatry A, Hedi Chaker Hospital 3029, Sfax, Tunisia

**Keywords:** Systemic sclerosis, sexual function, quality of life

## Abstract

**Introduction:**

Sexuality is an often neglected area in patients with rheumatic disease. The aim of this study is to assess sexual functioning and quality of life in a group of married women with Systemic Sclerosis (SSc).

**Methods:**

This is a horizontal study for descriptive and analytical purposes. Married women with SSc were interviewed about their sexual functioning and their quality of life.

**Results:**

A total of ten patients who met the criteria have accepted to participate to the study. Their mean age was 52, 4± 8,2 years. Eight women thought that the disease had affected their sexual activity. All patients reported a decrease in the frequency of intercourse since the onset of their disease. Eight of the sample reported a diminished desire for a sexual relationship. The reasons were fatigue, altered body image and pain. The assessment of sexual functioning using the Female sexual function index (FSFI) showed a mean FSFI score at 14,2±7,8 with nine women scoring in the range associated with sexual dysfunction (SD) (<26). All the subscales were affected. Our patients reported a mean total score on WHOQOL-brief (World Health Quality of Life-Brief Version) of 60 out of 120 indicating a moderate altered quality of life. Depression has been identified as determinants of impaired sexual function.

**Conclusion:**

The prevalence of SD in women with SSc is high when a specific questionnaire is used to assess it. These results indicate that in daily practice, inquiring about sexuality and screening for depressive symptoms is indicated for every patient with SSc.

## Introduction

Systemic Sclerosis (SSc) is a chronic autoimmune disease characterized by abnormal fibrotic processes, inflammation and microvascular damage [1]. Like the majority of connective tissue diseases, SSc has female predilection (approximately 80% of SSc patients are women). The vascular alterations and immunological activation lead to progressive and widespread fibrosis of multiple organ systems including the skin, the gastrointestinal tract and the lungs. Changes associated with SSc such as skin tightening, muscle weakness, joint pain, deformity and decreased physical function can have a negative impact on female sexuality and sexual functioning [1–3]. According to World Health Organization (WHO), sexual health is a state of physical, emotional, mental and social well-being in relation to sexuality [4]. This means that sexual health has to be seen from a holistic perspective, including physical, psychological, and social aspects of well-being. Sexuality is an important aspect of quality of life that is often neglected in research studies. In routine clinical practice, the focus is typically on fertility, pregnancy or contraception, whereas sexual function is not addressed [2, 5]. Some of the recent studies on female sexual functioning in SSc confirm the complexity of studying female sexual function as well as the psychological components of it [6–9]. Patients experience significantly impaired health-related quality of life compared with general population samples, and often report problems with sexual function. Sexual difficulties due to SSc can include decreased sexual arousal, decreased sexual desire, and decreased satisfaction. Problems that cause sexual health difficulties for women with SSc can be fatigue, pain, limited physical ability, negative body image, and depression. Our purposes were to assess the sexual functioning and the quality of life in a small sample of married women with SSc from our institution, to determine the etiological factors of sexual impairment and also to provide a review of the literature on sexual dysfunction among SSc patients.

## Methods

### Study design

We conducted a longitudinal study over a 9-months period between October 2011 and June 2012 at the Department of Internal Medicine in Sfax-Tunisia University Center. Patients were included if they were diagnosed with limited or diffuse SSc based on classification criteria of the American College of Rheumatology (ACR criteria) [10] and/or Leroy & Medsger [11] criteria for SSc. Eligibility for the study included only married women 18 years of age or older and who consented to participate. The choice of married women was made by the fact that musulman patients avoid talking about sexual health and sexual problems due to the sensitive nature of the topic and the concern that it could lead to embarrassment or discomfort. All women eligible were approached for participation in the study. Patients were evaluated on the same day by a physician and a psychiatrist.

### Sociodemographics and medical evaluation

Parameters recorded during medical evaluation were age (in years); year of onset of Raynaud ‘s phenomenon (RP); age at diagnosis; disease duration as measured from onset of the first non-Raynaud ‘s phenomenon symptom; disease form (limited SSc (lSSc) or diffuse cutaneous SSc (dSSc)), skin involvement evaluated by the modified Rodnan score; dyspnea (assessed by the New York Heart Association NYHA 4-point scale); and digital ulcers. The presence or absence of major scleroderma visceral involvement (esophageal-gastrointestinal, joint, muscle and/or heart involvement; interstitial lung disease (ILD); pulmonary arterial hypertension; and renal crisis) was recorded from clinical charts for hospitalized patients. Additional information on sociodemographics regarding education level, length of relationship, employment information, health problems, and medication use were extracted from medical records or obtained by patient-reported.

### Assessment methods

The women completed four instruments: the Health Assessment Questionnaire (HAQ) to evaluate Physical function [12], the Female Sexual Function Index (FSFI) to assess sexual function in the last 4 weeks [13], the abbreviated version of the World Health Organization Quality of Life (WHOQOL-BREF) and the Hospital Anxiety and Depression Scale (HADS) to assess patient ‘s mood. Completion of the 4 instruments took approximately 30 min.

### Disease activity, physical function and pain assessment

Descriptive data were compiled from the medical charts regarding the extent and severity of scleroderma. Patients were clinically examined to assess disease severity. Global disability was assessed using the HAQ which comprises 20 items divided into 8 domains and has been validated in French [12]. The scale ranges from 0 (no disability) to 3 (maximal disability). Pain was assessed with the single-item visual analogue scale (VAS). Patients place a mark on a 10-cm line at a point that corresponds with the intensity of their pain. Total scores of the VAS range from 0 to 100 (0 is labeled no pain and 100 is labeled worst possible pain).

### Assessment of sexual function

The age of first sexual problems related to the patient ‘s disease were discussed. In addition to other assessments, patients were asked by interview for their sexual status: the quality of sexual relationship, sexual satisfaction, sexual dysfunction, sexual interest and the relative frequency of sexual activity (whether or not it has increased or decreased). Patients completed some items related to problems that may be linked to sexual dysfunction in SSc. Satisfaction for sexual Relationship has been appreciated by choosing the most appropriate answer (dissatisfied, little satisfied, moderately satisfaction, very satisfied). To assess sexual function in the last 4 weeks, we used the FSFI standardized questionnaire [13]. The FSFI is relatively easy to administer and score and is currently being used in a number of clinical trials of female sexual dysfunction. It is a brief, 19-item self-report measure of female sexual function that provides scores on six domains of sexual function as well as a total score. The six domains include desire (2 items), arousal (4 items), lubrication (4 items), orgasm (3 items), satisfaction (3 items), and pain (3 items). Each question is given a score of 0 or 1 to 5. Each domain score and the total score are calculated. In addition, a total score (range 2- ‘36) can be computed. Higher subscale or total scores indicate better sexual function. A cutoff score of 26 is proposed as a criterion for impaired sexual function.

### Assessment of Quality of Life

Quality of life (QOL) was measured with the WHOQOL-BREF assessment, which includes 4 domains scores: physical health, psychologic health, social relationships, and environment [14]. It is a self-reported questionnaire that contains 26 items, and each item represents 1 facet. The facets are defined as those aspects of life that are considered to have contributed to a person ‘s QOL. Among those 26 items, 24 of them make up the 4 domains of physical health (7 items), psychologic health (6 items), social relationships (3 items), and environment (8 items), whereas the other 2 items measure overall QOL and general health. For statistical analysis, all domain scores were calculated by taking the mean score for all items included in each domain and transforming by a factor [15]. The score for each domain therefore ranged from 0 to 100, with a higher score indicating better QOL.

### Mental status

Anxiety and depression were measured by the Hospital Anxiety and Depression Scale (HADS), a 14-item questionnaire [16]. It was developed to indicate the possible presence of anxiety and depressive states in the setting of a medical outpatient clinic and is considered to be unbiased by coexisting general medical conditions. It contains two 7-items scales: one for anxiety (A) and one for depression (D). Each point is scored 0-3 and the total score ranges from 0 (no depression, no anxiety) to 21 (maximal depression, maximal anxiety). Scores of 0-7 in subscales are considered normal, 8-10 borderline and >11 clinical caseness [16].

### Need for counseling

At the end of the questionnaires, participants were asked if they felt the need to talk to someone about sexual issues, and if so, who they would confide in (their physician, their partner, a friend)

### 2.4. Statistical analysis

Data analysis was conducted using SPSS 11 Software. Descriptive statistics were calculated for all variables. Quantitative variables are described as the mean + standard deviation (SD) and range. Qualitative variables are described with proportions and percentages. P values (2-sided) less than 0.05 were considered statistically significant. The univariate association between sexual functioning and sexual distress with somatic and psychological characteristics was assessed with Pearson ‘s, Spearman ‘s, or point-biserial correlation coefficients as appropriate.

## Results

### Demographic and clinical data of the sample

For the entire longitudinal study, 10 of 30 patients approached in the clinic were married and agreed to participate. The percentage of patients who were married was lower than that for their siblings. The epidemiological characteristics and some clinical features of the studied patients are shown in Table 1. Their mean age at the time of evaluation was 52.4± 8.2 years (ranging from 38 to 65 years). Six women completed at least some college; and 5 reported that they were disabled and unable to work. The mean age at the marriage was 26.3±8.3 years (18-41 years). The mean age at disease onset was 45.2 ± 14 (18-65 years) and the mean disease duration was 7.7 ± 7.7 years. A total of 7 had dSSc and 3 had lSSc. The mean modified Rodnan score was 9.5 ± 6.6 (range 2-22). Eight patients had RP: 6 moderate RP and 2 severe RP. Eight women had stomach problems, two had hypertension, 1 heart problems, 1 thyroiditis, and 2 had ILD. Almost all patients were taking medication for their SSc irrespective of possible side effects for sexual functioning (Table 1). The percentage of patients with children was 90% (9/10) with an average of 3.3 children per family.

**Table 1:** Demographic, clinical characteristics, disability of patients with systemic sclerosis (SSc)

Parameters	SSc patients (n = 10)
Age ( mean + SD years)	52.4± 8.2
Limited SSc	3
Diffuse SSc	7
Disease duration (mean +SD years)	7.7 ± 7.7
Modified Rodnan skin score (mean + SD)	9.5 ± 6.6
Raynaud phenomenon	8
Esophageal-gastrointestinal involvement	8
**NYHA**	
0	2
I	3
II	2
III	3
IV	0
Digital ulcers	1
Arthralgia	9
ILD	2
HAQ (mean + SD)	1.15 + 0.5
**Medication**	
Analgesics (including NSAID)	1
Colchicine	10
D-penicillamine	2
Corticosteroid (10 mg/day)	3
Calcium channel blockers	3
Proton-pump inhibitor	8

SSc: systemic sclerosis; NYHA: New York Health Association; ILD: interstitial lung disease; HAQ: Health Assessment Questionnaire; NSAID: non steroid anti-inflammatory drugs

### Outcome measure scores

The mean VAS score for body pain was 64 ± 20 mm (range: 30- 100 mm). Body pain was judged high (VAS score > 60 mm) in six patients. The mean HAQ global disability score was 1.15 ± 0.5 (0.25-1.87). Eight women thought that the disease had affected their sexual relationship and their sexual activity. All patients reported a decrease in the frequency of intercourse since the onset of their disease. The reported mean age at the beginning of sexual dysfunction (SD) was 47.3 ± 11.5 years (32 - 65 years). Eight of the sample reported a diminished desire for a sexual relationship since the disease onset. The major reasons were fatigue (8 cases), altered body image (7 cases) and pain (8 cases). Other scleroderma-related problems were identified by participants as contributing to sexual difficulties such as vaginal dryness (10 cases), dyspareunia (2 cases), joint pain (3 cases) and joint stiffness (2 cases). Two patients reported don't having engaged in sexual activities with her partner in the past 4 weeks. The satisfaction about sexual relationship was considered as altered for 8 women. Three women were very dissatisfied, 3 were little satisfied, and 2 were moderately satisfied. Only 2 women reported that they were globally satisfied with their sexual status. The mean FSFI score in our population was 14.2 ±7.8 (SD = 7.8, range = 2-28) which is significantly lower than the mean score of 30.5 reported for the general population. Nine women scoring in the range associated with clinical SD (<26). All the subscales were affected: desire, arousal, lubrication, orgasm and satisfaction. FSFI total scores and scores of each domain are shown in Table 2. Regarding the WHOQOL-BREF Assessment, the mean scores were 46 (SD= 8,8, range = 31-56) on the physical health domain; 50.7 (SD= 11.2, range= 31-69) on the psychologic health domain; 57.5 (SD= 15,5, range = 25-75) on the social relationships domain and 52 (SD= 11, range = 31-63) on the environment domain (Table 3). Our patients reported a mean total score on WHOQOL-brief (World Health Quality of Life-Brief Version) of 60 out of 120 indicating a moderate altered quality of life. The mean (+SD) HAD A score was 12.6± 3.4 (7-18) and the mean (+SD) HAD D score was 11.2 ± 5.8 (1-20) (Table 4). Eight patients had HAD A score > 11 corresponding to clinical anxiety and 7 patients had HAD D score > 11 corresponding to clinical depression. Univariate analysis indicated that disease duration, pain, psychological characteristics and depression were significantly associated with the FSFI total score (P < 0.05). Non significant relationships were found between FSFI global score and age, marriage duration, Rodnan score or HAQ score. In our own studies of quality of female sexual function, we found stronger correlations with the psychological component of the WHOQOL-BREF (p = 0,01) than with the physical component score (p = 0,38). The FSFI score was significantly lower for patients with current depression (p = 0.03) (Table 5).

**Table 2:** Scores for the FSFI

Female Sexual function index (FSFI)	Mean score ± SD (range)(n = 10)
Desire domain	1.9±0.6 (1.2-3)
Arousal domain	2.1±1.4 (0-4.5)
Lubrication domain	2.5±1.7 (0-4.8)
Orgasm domain	2.5±1.6 (0-5.6)
Satisfaction domain	2.5±1.3 (0.8-5.6)
Pain domain	2.4±1.6 (0-4.8)
Total instrument	14.2±7.8 (2-28)

**Table 3:** Domain Structure of the WHOQOL-BREF Assessment

Domaines du WHOQOL-BREF	Mean score+ SD (range)
Physical Health domain (0-100)	50±10 (38-63)
Psychologic Health domain (0-100)	39±11,6 (19-56)
Social Relationships domain (0-100)	54±22,2 (19-75)
Environment domain (0-100)	50,3±17,7 (31-88)

**Table 4:** Scores on the two subscales of Hospital Anxiety and Depression Scale (HAD A, anxiety and HAD D, depression)

HAD scale	Mean score+ SD (range)
Anxiety subscale (HAD A)	12,6± 3,4 (7-18)
Depression subscale (HAD D)	11,2±5,8 (1-20)
HAD	23,8±9 (10-38)

**Table 5:** Correlation of the FSFI total score with sociodemographic variables, quality of life, physical and psychological functioning

	FSFI (n = 10)
Age (years)	r = -0,43; p= 0,8
Disease duration	r =0,6; p = 0,02
Marriage duration	r = -0,48; p =0,08
Rodnan score	r = -0,26; p =0,17
HAQ score	r = -0,32; p = 0,26
Pain scale	r = 0,1; p = 0,014
**WHOQOL-BREF**	
Physical health domain	r = 0,34 (p = 0,38)
Psychological health domain	r = 0,4 (p = 0,01)
Social domain	r = 0,69 (p = 0,4)
Environment domain	r = 0,35 (p = 0,2)
**HADS**	
Anxiety	r = -0,54 (p = 0,2)
Depression	r = -0,7 (p = 0,03)

r = correlation coefficient; p : probability ( significant if <0,05)

### Need for counseling

Seven patients (70%) with SSc reported a need to talk to someone about current sexual problems.

## Discussion

Our results show a high prevalence of SD among married women with SSc. However, there are some limitations in this study that should be considered. The small size of the SSc sample and the non comparison with a healthy group suggest caution in interpreting results and could influence generalizability.

Female sexual dysfunction (FSD) is defined by the American Foundation of Urological Disease as a diminished or absent feeling of sexual interest or desire, absent sexual thoughts or fantasies and a lack of responsive desire. FSD includes sexual desire disorder, sexual arousal disorder, female orgasmic disorder, and sexual pain disorders [17]. The National Health and Social Life Survey suggested that 43% of all women are affected by some form of FSD [18, 19]. This frequency is substantially higher in women dealing with chronic diseases [19]. There is an abundance of research into the effects on sexuality of chronic diseases such as diabetes and renal disease, but there is relatively little pertaining to sexuality and connective diseases (SLE, SS, SSc). The number of female patients with SSc reporting SD is higher than those reported in the general population and also higher than those reported in studies on other chronic diseases [2, 6, 7, 20]. A recent systematic review found that women with diffuse or limited SSc experienced levels of sexual impairment similar to or higher than women with breast cancer, HIV and gynaecological cancer [2]. Bhadauria et al [20] compared sexual function between 60 patients with SSc and 23 women with rheumatoid arthritis (RA) or SLE matched for age and disease duration. It was found that women with SSc had a significantly lower number and intensity of orgasms and significantly more women with SSc reported skin tightness and heartburn, reflux, or vomiting as symptoms causing problems during intercourse compared with the control subjects. Most female SSc patients are sexually inactive because of complications related to their disease. Impens et al. [6] reported that 17% of patients were sexually inactive because of issues caused by their disease. In a survey by Guerriere et al, 40% of patients with SSc reported being sexually inactive, with only 7% attributing their sexual inactivity to SSc [21]. In spite of the 80% female predominance of SSc, most of the studies on the effect of SSc on sexual function have involved men [5, 20, 22–27]. Little is known of the impact of scleroderma on female sexual functioning. The complexity and multifactorial nature of female sexual response and female sexual dysfunction adds to the difficulty in research in this area. SSc is responsible for skin, tendon, joint, and vessel damage, which leads to disability and handicap [12]. As a consequence of skin sclerosis and telangiectasias, SSc patients may experience morphological changes prominently localized to the hands and face. Early disease is mediated through microvascular dysfunction secondary to a number of factors including endothelial damage, over expression of specific adhesion molecules, and perivascular inflammatory cell infiltration [28].

The ability to achieve healthy sexual function involves both physiological factors that affect the sexual response cycle and interpersonal factors that influence intimate partner relationships. For women with SSc, both physiological and psychosocial factors often result in impaired sexual function. Physical problems, emotional problems and partnership difficulties arising from disease-relatedness contribute to a less active and often less enjoyable sex life [29]. Chronic pain, fatigue and low self-esteem can reduce an individual's sexual interest and thereby reduce the frequency of intercourse. The pleasure of intercourse can become diminished by pain of joint movement, or difficulty in finding positions that do not cause discomfort. Saad et al [23] have documented that skin involvement, Raynaud's phenomenon, gastrointestinal symptoms, joint pain and vaginal dryness with dyspareunia contribute to pain and discomfort during sexual activity for women with SSc. Tightening of facial skin with shrinking of the mouth and finger ulcers can also impede sexual activity [23]. Weaver and Byers demonstrated that women's sexual problems are closely linked to the idea they have of their bodily appearance. Many women become sexually aroused by finding themselves desirable to their partner. It is therefore more difficult for a woman who does not consider herself attractive to understand that another person could find her desirable [30].

Fatigue is a common symptom of SSc that decreases quality of life by diminishing the ability to engage in meaningful personal and social activities and has important implications for employment, compliance with medical treatments and the use of healthcare services [31]. Many patients with SSc have significant limitations on exercise capacity with dyspnea, decreased stamina, and coughing that may interfere with certain sexual behaviours [23, 24]. Sexual pain disorders are additional major contributors to sexual dysfunction in this population. Pain is caused by vaginal dryness and leads to avoidance of intimacy, and fear of intercourse. Saad et al. [5] found that 45% of 65 women with SSc and SS reported vaginal dryness that can make sexual intercourse uncomfortable. Another study found that nearly one-third of 150 female SSc patients reported dyspareunia based on a single-item inquiry [32]. Our study demonstrated a lower frequency of sexual intercourse, orgasm and decreased sexual desire in those women following the onset of SS. FSD was significantly correlated to disease duration and body pain. Although the majority of sexual problems were related to physical disability and pain, there was a high incidence of psychological problems concerning self-confidence and perceived attractiveness. Thombs et col. reported that about 36 - 65% of the SSc patients have clinically significant symptoms of depression [33]. Roca et col found in his study that sexual dysfunction was more closely associated with symptoms of depression than any other functional domain [34]. Two studies found that FSFI scores were unrelated to disease classification and that women with limited SSc reported as many problems with SF as patients with diffuse disease. No relationship between disease severity and sexual function was found, whereas depressive symptoms were highly present [7, 21]. Those findings are in accordance to our study in which we found that psychological characteristics and more depressive symptoms were significantly associated with sexual dysfunction (P < 0.05).

## Conclusion

Our findings add to the existing literature an important aspect of the sexual and psychological complications of SSc. In addition to the emotional and psychological burden that SSc places on female patients, there is an additional physical burden which may impact sexual functioning and further decrease quality of life. Previous studies documented that women with SSc do remain sexually active overall in spite of several disease-related physical and psychological difficulties. Many of their problems are amenable to health interventions and should be addressed during health care visits. Our results lead to call attention to the role of psychological health in individuals with chronic disease such as SSc thus suggesting additional opportunities for interventions to enhance quality of life.
